# Relative Bioavailability of Fentanyl Following Various Dosing Regimens of Fentanyl Buccal Tablet in Healthy Japanese Volunteers

**DOI:** 10.1111/j.1753-5174.2008.00009.x

**Published:** 2008-09

**Authors:** Mona Darwish, Kenneth Tempero, John G Jiang, Philip G Simonson

**Affiliations:** * Cephalon, Inc.Frazer, PA, USA; † 1901 Lake Road, Wayzata, MN, USA

**Keywords:** Fentanyl Buccal Tablet (FBT), Bioavailability, Pharmacokinetics, Japanese

## Abstract

**Background:**

Fentanyl buccal tablet (FBT; *FENTORA*®, Cephalon, Inc., Frazer, PA, USA) is indicated in the US for breakthrough pain in patients with cancer who are already receiving and are tolerant to around-the-clock opioid therapy for underlying persistent cancer pain. For each individual patient, FBT should be titrated to the effective dose.

**Objective:**

The primary objective was to characterize the pharmacokinetic parameters of FBT 400 µg administered as a single 400 µg tablet (regimen A) or as two 200 µg tablets given simultaneously (regimen B) and determine whether these are bioequivalent in healthy Japanese volunteers. Regimen C (two 200 µg tablets 30 minutes apart) was also compared as a secondary objective.

**Methods:**

Healthy Japanese adults received regimens A, B, and C in a crossover fashion. Naltrexone was given to minimize the opioid effects of fentanyl. Serum fentanyl concentrations were determined in venous blood collected through 36 hours post dose. Regimens were declared bioequivalent with respect to bioavailability (as reflected by AUC_0–∞_, AUC_0–last_, and C_max_) if the 90% confidence interval (CI) of the regimens' ratio fell within 0.80–1.25 (80%–125%).

**Results:**

Twenty-nine volunteers (13 men, 16 women) were enrolled; 24 completed the study. Regimens A and B had bioequivalent systemic exposure parameters (B/A [90% CI]: AUC_0–∞_108.4 [103.4, 113.7], AUC_0–last_ 106.1 [100.7, 111.7], and C_max_ 92.3 [83.2, 102.4]). Regimen C was bioequivalent to both A and B for AUCs, but only to B for C_max_. Median time to C_max_ was 45 minutes for regimen A and 60 minutes for regimens B and C. The most frequent AEs were dizziness, application-site erythema, headache, somnolence, nausea, and vomiting. All AEs were mild or moderate.

**Conclusions:**

Bioavailability of fentanyl after FBT 400 µg administered as a single tablet was bioequivalent to that after 2 simultaneously administered 200 µg tablets in healthy Japanese volunteers. AEs were mild or moderate.

## Introduction

Fentanyl buccal tablet (FBT; *FENTORA*®, Cephalon, Inc., Frazer, PA, USA) is a novel formulation of fentanyl that uses OraVescent® technology (CIMA LABS, INC., Eden Prairie, MN, USA) to enhance the rate and extent of fentanyl absorption across the buccal mucosa [1]. FBT has been shown to provide onset of analgesia in 10 minutes and is indicated for the management of breakthrough pain in patients with cancer who are already receiving and are tolerant to around- the-clock opioid therapy for underlying persistent cancer pain [2–4].

FBT is available in doses of 100, 200, 300, 400, 600, and 800 µg, and it should be titrated to the effective dose for each individual patient. To facilitate titration, it would help to understand the relative bioavailability achieved with multiple tablets of a low dose compared with that achieved with a single tablet of a higher dose. This information may allow the patient to switch from multiple low-dose tablets to a single higher-dose tablet if necessary [5].

This study was conducted to support the submission of a new drug application for FBT in Japan. The primary objective of the study was to characterize the pharmacokinetic parameters of FBT 400 µg administered as a single 400 µg tablet (regimen A) or as two 200 µg tablets administered simultaneously (regimen B) and to determine whether these 2 regimens provide bioequivalent bioavailability (as reflected by parameters measuring systemic exposure) in healthy Japanese volunteers. Secondary objectives included evaluation of the pharmacokinetic parameters of FBT 400 µg administered as two 200 µg tablets given 30 minutes apart (regimen C) and comparison of the results with those obtained with regimens A and B.

## Methods

### Study Population

Healthy Japanese men and women residing in the United States who were aged 20 to 55 years, had lived fewer than 10 years outside of Japan, and had a body mass index of 17.6 to 29 kg/m^2^ were eligible for the study. Inclusion criteria included clinically normal findings on physical examination and from medical history (no cardiovascular, pulmonary, hepatic, renal, hematologic, gastrointestinal, endocrine, immunologic, dermatologic, neurologic, or psychiatric disease). Results from laboratory tests and electrocardiography were required to be within normal ranges.

Prior to the first dose of study drug, volunteers were not to have taken the following agents: prescription medications (except for oral contraception for women) within 14 days, over-the-counter medication and herbal supplements within 7 days, strong inhibitors of cytochrome P450 (CYP) enzymes within 10 days, and strong inducers of CYP enzymes within 30 days. These agents were not allowed during the study.

Volunteers were excluded if they had smoked more than 10 cigarettes per day during the 3 months prior to the first dose of FBT. Tobacco use was limited to ≤10 cigarettes during the washout phases. Women who were pregnant or lactating were excluded from the study.

### Study Design

This open-label, randomized, 3-phase crossover study was conducted at a single center (Radiant Research, Honolulu, HI). Aspire Institutional Review Board approved the study protocol. All volunteers provided written informed consent.

Volunteers were randomly assigned to receive FBT 400 µg as a single-dose tablet (regimen A), two 200 µg tablets administered simultaneously (regimen B), and two 200 µg tablets administered 30 minutes apart (regimen C) in a crossover design with a 7-day washout between regimens. For regimens B and C, 1 tablet was placed on each side of the mouth. To minimize carryover effects, regimens were allocated using a Latin square design. Because healthy volunteers are not opioid tolerant, naltrexone was given during each regimen to minimize the opioid effects of fentanyl; they received 1 naltrexone 50 mg tablet 15 hours and 3 hours before and 12 hours after FBT placement. Coadministration of naltrexone with fentanyl would not be expected to affect the pharmacokinetics of fentanyl because fentanyl is a substrate of CYP3A4 [6], and naltrexone is not an inhibitor or inducer of CYP3A4 [7].

Volunteers self-administered FBT by placing it above a molar tooth (i.e., buccally) and allowing it to dissolve for 10 minutes. If any portion of the tablet remained, volunteers were instructed to gently massage the adjacent cheek area for 5 minutes to facilitate dissolution. Any tablet residue remaining at this time (verified by study center personnel) was allowed to dissolve on its own (further massaging of the cheek area by the subject was allowed, if needed). FBT was administered at the same time of day in each study phase.

### Dwell Time Assessment

Dwell time, defined as the time from tablet placement to complete disappearance of any tablet residue (verified visually), was recorded by study personnel.

### Sample Collection

Venous blood was collected immediately before and 5, 10, 15, 20, 25, 30, 35, 40, 45, 50, and 55 minutes, and 1, 1.25, 1.5, 1.75, 2, 2.5, 3, 4, 6, 8, 10, 12, 16, 20, 24, 28, 32, and 36 hours after FBT placement. Blood samples were allowed to clot at room temperature. The serum was separated by centrifugation at 2,500 revolutions per minute for 15 minutes at 4°C, then stored in polypropylene containers at or below −20°C until assayed.

### Analytical Methods

Serum fentanyl concentrations were quantified using a validated assay for high performance liquid chromatography-mass spectrometry/mass spectrometry (HPLC-MS/MS; PE SciEx API 3000, API 4000, API 365, and API III Plus with an ESI interface). The analyte and internal standard (d_5_-fentanyl) were isolated by liquid-liquid extraction under basic conditions. Serum extracts were evaporated to dryness and reconstituted for injection onto the HPLC-MS/MS. Detection was accomplished using multiple-reaction monitoring in positive ion mode. The assay was linear from 10 pg/mL to 5,000 pg/mL (0.01 to 5.0 ng/mL), with a lower limit of quantitation of 50 pg/mL (0.05 ng/mL). Serum concentrations below the limit of quantitation were assigned a value of zero. Quality control samples had an interbatch precision (% coefficient of variation) of ≤3.6% and an interbatch accuracy (% bias) ranging from 100.3% to 101.3%.

### Pharmacokinetic Analysis

The pharmacokinetic parameters determined included maximum serum fentanyl concentration (C_max_), time to C_max_ (t_max_), area under the serum fentanyl concentration-time curve (AUC) from time zero (t_0_) to the time of the last quantifiable serum concentration (t_last_) (AUC_0–last_), AUC from t_0_ extrapolated to infinity (AUC_0–∞_), the terminal elimination phase rate constant (λ_z_), and the elimination half-life (t_½_). AUC_0–last_ was obtained by linear trapezoidal summation from t_0_ to t_last_, and AUC_0–∞_ was obtained as (AUC_0–last_ + C_last_/λ_z_), where C_last_ was the last quantifiable serum fentanyl concentration. The λ_z_ and t_½_ values were obtained from linear regression of the terminal portion of the log concentration-versus-time curve. The pharmacokinetic parameters obtained were used to compare regimens A and B, B and C, and A and C. Pharmacokinetic analysis was performed using standard noncompartmental methods with WinNonlin® Professional software Version 4.1 or higher (Pharsight Corp., Mountain View, CA).

### Safety and Tolerability Assessments

Clinical laboratory tests, a physical examination with vital signs, and 12-lead electrocardiography were performed at the screening visit and on completion of the study or at the time of early termination. Serial vital signs were recorded through 36 hours following each study drug administration, and continuous pulse oximetry was performed for the first 4 hours post dose, as well as any time the volunteers attempted to sleep when they were in the clinic. Adverse events (AEs), including any uncomfortable or unpleasant sensations in the mouth, were recorded. Study personnel examined the oral mucosa at the site of tablet placement 1 hour after FBT administration (in the case of regimen C, after the second tablet).

### Statistical Analyses

A sample size of 22 volunteers was anticipated to provide 90% power to reject the null hypothesis of nonbioequivalence between regimens A and B using the 2 one-sided tests procedure at the 0.05 level. An analysis of variance model was performed on the log-transformed values for C_max_, AUC_0–∞_, and AUC_0–last_, with fixed effects for regimen, phase, and regimen order, and subject nested within regimen order as a random effect. The 90% confidence interval (CI) for the difference in the regimens' least square geometric means from this model was then exponentiated to yield a 90% CI for the ratio of the regimens' least square geometric means. Bioequivalence was concluded if the 90% CIs of the ratios of the regimens' least square geometric means for AUC_0–∞_, AUC_0–last_, and C_max_ fell within the interval of 0.80 to 1.25 (80%–125%).

Pharmacokinetic, safety, and tolerability parameters were summarized using descriptive statistics; tests were 2-sided with an α level of 0.05. Wilcoxon signed rank testing was used to analyze t_max_.

The evaluable analysis set comprised the randomized volunteers who completed at least 2 periods of the study; this set was used for the analysis of bioequivalence. The pharmacokinetic analysis set comprised the randomized volunteers who received at least 1 dose of FBT and had sufficient serum fentanyl concentration data to allow calculation of at least 1 pharmacokinetic parameter. The safety analysis set comprised the randomized volunteers who received at least 1 dose of FBT; the safety analysis set was used for population summaries, unless otherwise noted.

## Results

### Study Population

Twenty-nine Japanese volunteers (13 men, 16 women) aged 20 to 50 years were enrolled in the study (Table 1) and all 29 were included in both the safety and pharmacokinetic populations. The evaluable population for the bioequivalence analyses comprised 26 patients. Twenty-seven volunteers completed regimens A and B, and 26 completed regimen C. Twenty-four volunteers completed the study; 2 were discontinued because of AEs and 3 others withdrew their consent to participate.

**Table 1:** Demographic variables

	N = 29
Age, year (mean [SD])	32 (8)
Sex, female (N [%])	16 (55.2)
Weight, kg (mean [SD])	60.4 (11.4)
Height, cm (mean [SD])	165.2 (11.4)
Body mass index, kg/m^2^ (mean [SD])	22.0 (2.6)

SD = standard deviation.

### Pharmacokinetic Parameters

**Figure 1:**
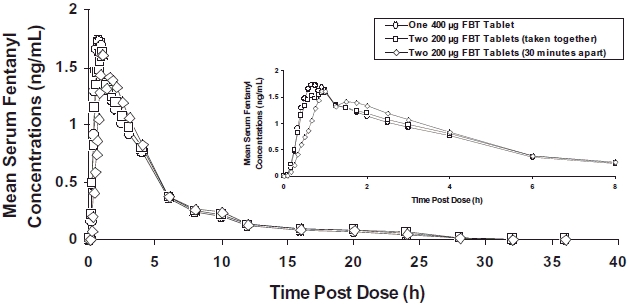
Mean serum fentanyl concentrations after fentanyl buccal tablet administration delivered as one 400 µg tablet (regimen A), two 200 µg tablets simultaneously (regimen B), or two 200 µg tablets administered 30 minutes apart (regimen C). Inset is an expanded view of the first 8 hours after drug administration.

The concentration-time profiles for fentanyl are shown in Figure 1. After placement of FBT, regimens A, B, and C were characterized by a rapid absorption phase followed by a triexponential decline from peak concentration. Pharmacokinetic parameters are listed in Table 2. Median t_max_ was 45 minutes for regimen A and 60 minutes for regimens B and C (Table 2).

**Table 2:** Pharmacokinetic parameters of fentanyl following various dosing regimens for 400 µg of fentanyl buccal tablet

	Regimen, mean (SD)		
	A: One 400 µg tablet	B: Two 200 µg tablets simultaneously	C: Two 200 µg tablets 30 minutes apart
Parameter	(N = 27)	(N = 27)	(N = 26)
C_max_ (ng/mL)	2.18 (0.60)	2.05 (0.70)	1.89 (0.54)
t_max_* (min)	45 (20, 105)	60 (25, 182)	60 (45, 150)
AUC_0–∞_ (ng·h/mL)	8.54 (2.29)	8.91† (2.17)	8.97 (2.25)
AUC_0–last_ (ng·h/mL)	7.88 (2.13)	8.16 (1.94)	8.29 (2.06)
λ_z_ (1/h)	0.11 (0.05)	0.10† (0.05)	0.10 (0.03)
t_½_ (h)	7.63 (3.52)	8.55† (3.47)	7.71 (2.55)

* Data presented as median (range).

† N = 26.

AUC_0–∞_ = area under the serum fentanyl concentration-time curve (AUC) from time zero extrapolated to infinity; AUC_0–last_ = AUC_0_ to the time of the last quantifiable serum fentanyl concentration; C_max_ = maximum serum fentanyl concentration; λ_z_ = terminal elimination phase rate constant; t_max_ = time to C_max_; t_½_ = elimination half-life.

The AUC_0–last_ and AUC_0–∞_ values were bioequivalent between regimens A and B, regimens B and C, and regimens A and C because the 90% CIs for the regimens' ratios were contained entirely within the predefined limits of 0.80 to 1.25 (Table 3). The C_max_ values were bioequivalent between regimens A and B and between regimens B and C. Regimens A and C did not show a bioequivalent C_max_ because the lower limit of the 90% CI of the ratio was marginally outside the predefined lower limit allowed by the protocol (Table 3).

**Table 3:** Statistical analysis for bioequivalence following various fentanyl buccal tablet 400 µg dosing regimens

		Mean†	Ratio over A	Ratio over B
Parameter	Regimen*	(N = 26)	% (90% CI)	% (90% CI)
AUC_0–∞_ (ng·h/mL)	A	8.241	—	—
	B	8.937	108.4 (103.4, 113.7)	—
	C	8.790	106.7 (101.7, 111.8)	98.4 (93.9, 103.1)
AUC_0–last_ (ng·h/mL)	A	7.620	—	—
	B	8.083	106.1 (100.7, 111.7)	—
	C	8.123	106.6 (101.2, 112.2)	100.5 (95.5, 105.7)
C_max_ (ng/mL)	A	2.146	—	—
	B	1.981	92.3 (83.2, 102.4)	—
	C	1.833	85.4 (77.0, 94.7)	92.5 (83.6, 102.5)

* Fentanyl buccal tablet study regimens: regimen A = one 400 µg tablet; regimen B = two 200 µg tablets administered simultaneously; regimen C = two 200 µg tablets administered 30 minutes apart.

† Values for AUC and C_max_ are least squares geometric means.

AUC_0–∞_ = area under the serum fentanyl concentration-time curve (AUC) from time zero extrapolated to infinity; AUC_0–last_ = AUC_0_ to the time of the last quantifiable serum fentanyl concentration; CI = confidence interval; C_max_ = maximum serum fentanyl concentration.

### Buccal Dwell Time

The mean dwell time of FBT in the buccal cavity was 51.41 minutes following regimen A (range 12–110 minutes), 67.96 minutes followingregimen B (range 15–149 minutes), 50.85 minutes following placement of the first tablet in regimen C (range 12–118 minutes), and 64.12 minutes following placement of the second tablet in regimen C (range 13–151 minutes).

### Safety and Tolerability

As stated previously, naltrexone was administered to these healthy volunteers to minimize the opioid effects of fentanyl. Twenty-three volunteers (79.3%) had at least 1 AE, with all AEs reported as mild or moderate. No serious AEs occurred during the study. Two volunteers were discontinued from the study because of AEs that occurred after administration of the first dose of naltrexone: one because of nausea and vomiting, and another because of vomiting and dizziness. In both cases, the AEs occurred prior to receipt of FBT and were considered by the investigator not to be related to FBT.

Table 4 displays the most common AEs for each dosing regimen. Application-site AEs, all of which were mild, occurred after administration of the 200 µg tablets (regimens B and C), but not after administration of the 400 µg tablet (regimen A). There were no clinically relevant changes in vital signs or electrocardiographic measurements. One volunteer had a newly diagnosed finding (rash) at the end-of-study physical examination that was considered by the investigator to be mild and not related to study drug. Laboratory abnormalities considered clinically significant were reported for 1 volunteer (hyperglycemia and glucosuria) who had the measurements taken approximately 2 to 3 hours after dinner (postprandial).

**Table 4:** Adverse events occurring in ≥5% of volunteers in any one of various fentanyl buccal tablet 400 µg dosing regimens*

	Regimen A: One 400 µg tablet	Regimen B: Two 200 µg tablets simultaneously	Regimen C: Two 200 µg tablets 30 minutes apart	Total
	(N = 27)	(N = 27)	(N = 26)	(N = 29)
Adverse event	N (%)	N (%)	N (%)	N (%)
Application-site AEs				
Erythema	0	4 (14.8)	4 (15.4)	7 (24.1)
Ulcer	0	0	2 (7.7)	2 (6.9)
Dizziness	2 (7.4)	5 (18.5)	2 (7.7)	8 (27.6)
Headache	3 (11.1)	3 (11.1)	2 (7.7)	6 (20.7)
Nausea	0	3 (11.1)	2 (7.7)	5 (17.2)
Somnolence	4 (14.8)	5 (18.5)	3 (11.5)	6 (20.7)
Vomiting	0	1 (3.7)	2 (7.7)	3 (10.3)

* Volunteers who had multiple episodes of a given adverse event were counted once.

## Discussion

The primary purpose of this study was to evaluate the pharmacokinetic parameters and the relative bioavailability of FBT administered as a single 400 µg tablet (regimen A) and as two 200 µg tablets administered simultaneously (regimen B) in healthy Japanese volunteers who were administered naltrexone to minimize opioid-receptor–mediated effects of fentanyl. Systemic exposure to fentanyl, reflected by AUC_0–∞_, AUC_0–last_, and C_max_, was bioequivalent with these 2 dosing regimens. Systemic exposure (AUC_0–∞_, AUC_0–last_, and C_max_) was also bioequivalent with 2 FBT 200 µg tablets administered simultaneously (regimen B) and 2 FBT 200 µg tablets administered 30 minutes apart (regimen C). FBT administered as a single 400 µg tablet and as two 200 µg tablets administered 30 minutes apart was bioequivalent with respect to AUC_0–∞_ and AUC_0–last_, but not with respect to C_max_, which was lower because volunteers waited 30 minutes to administer the second 200 µg dose in regimen C. Median t_max_ values ranged from 45 to 60 minutes among the 3 regimens.

The mean dwell times of FBT in the buccal cavity ranged from 50.8 to 68.0 minutes. It has already been shown that the pharmacokinetic profile of fentanyl delivered by FBT is not affected by variations in the dwell time of FBT [8].

All AEs were mild or moderate and no serious AEs occurred. The AE profile was similar to that found in previously published studies of opioid-naive volunteers who were administered naltrexone [5,9–12]. It should be noted that interpretation of the non–application-site AEs is difficult because of the concomitant administration of naltrexone. The incidence of application-site AEs suggests that oral mucosal changes were more frequent with the use of 2 FBT 200 µg tablets than with a single FBT 400 µg tablet, perhaps because of the additional mucosal exposure.

One must use caution in applying the results of this study to the clinical setting. Healthy volunteers are not representative of patients with breakthrough pain. The presence of comorbid conditions, such as hepatic or renal failure, may influence the concentrations of fentanyl achieved. Furthermore, the volunteers in this study were administered naltrexone to minimize the opioid effects of fentanyl. Thus, the safety and tolerability results may not be generalized to a patient population.

In conclusion, in healthy Japanese volunteers, systemic exposure to fentanyl was bioequivalent with 2 FBT 200 µg tablets administered simultaneously and a single FBT 400 µg tablet. AEs were mild or moderate.

## References

[1] Durfee S, Messina J, Khankari R (2006). Fentanyl effervescent buccal tablets. Am J Drug Deliv.

[2] Portenoy RK, Taylor D, Messina J, Tremmel L (2006). A randomized, placebo-controlled study of fentanyl buccal tablet for breakthrough pain in opioid-treated patients with cancer. Clin J Pain.

[3] Slatkin NE, Xie F, Messina J, Segal TJ (2007). Fentanyl buccal tablet for relief of breakthrough pain in opioid-tolerant patients with cancer-related chronic pain: A double-blind, randomized, placebo-controlled study. J Support Oncol.

[4] (2007). Fentora [prescribing information.

[5] Darwish M, Kirby M, Robertson P, Hellriegel E, Jiang JG (2006). Comparison of equivalent doses of fentanyl buccal tablets and arteriovenous differences in fentanyl pharmacokinetics. Clin Pharmacokinet.

[6] Tateishi T, Krivoruk Y, Ueng YF, Wood AJ, Guengerich FP, Wood M (1996). Identification of human liver cytochrome P-450 3A4 as the enzyme responsible for fentanyl and sufentanil *N*-dealkylation. Anesth Analg.

[7] Adams M, Pieniaszek HJ, Gammaitoni AR, Ahdieh H (2005). Oxymorphone extended release does not affect CYP2C9 or CYP3A4 metabolic pathways. J Clin Pharmacol.

[8] Darwish M, Kirby M, Jiang JG (2007). Effect of buccal dwell time on the pharmacokinetic profile of fentanyl buccal tablet. Expert Opin Pharmacother.

[9] Darwish M, Tempero K, Kirby M, Thompson J (2006). Relative bioavailability of the fentanyl effervescent buccal tablet (FEBT) 1,080 µg versus oral transmucosal fentanyl citrate 1,600 µg and dose proportionality of FEBT 270 to 1,300 µg: A single-dose, randomized, open-label, three-period study in healthy adult volunteers. Clin Ther.

[10] Darwish M, Kirby M, Robertson P, Tracewell W, Jiang JG (2006). Pharmacokinetic properties of fentanyl effervescent buccal tablets: A phase I, open-label, crossover study of single-dose 100, 200, 400, and 800 µg in healthy adult volunteers. Clin Ther.

[11] Darwish M, Kirby M, Robertson P, Hellriegel E, Jiang JG (2007). Single-dose and steady-state pharmacokinetics of fentanyl buccal tablet in healthy volunteers. J Clin Pharmacol.

[12] Darwish M, Kirby M, Robertson P, Tracewell W, Jiang JG (2007). Absolute and relative bioavailability of fentanyl buccal tablet and oral transmucosal fentanyl citrate. J Clin Pharmacol.

